# An increasing number of hand injuries in an elderly population – a retrospective study over a 30-year period

**DOI:** 10.1186/s12877-018-0758-7

**Published:** 2018-03-09

**Authors:** Hans-Eric Rosberg, Lars B. Dahlin

**Affiliations:** 1 Department of Hand Surgery, Skåne University Hospital, Jan Waldenströms gata 5, SE-205 02 Malmö, Sweden; 2 Translational Medicine - Hand Surgery, Lund University, Jan Waldenströms gata 5, SE-205 02 Malmö, Sweden

**Keywords:** Hand injury, Elderly

## Abstract

**Background:**

Both the number and the proportion of elderly people in the society increase. The number of elderly subjects with a disability due to a disease has decreased resulting in more active elderly. Therefore, an increase in numbers of injury in the elderly population can be expected; a hypothesis that was investigated in the present study.

**Methods:**

Two-hundred sixteen patients with an age of > 65 years, and admitted to a hand surgery ward with a hand injury, were retrospectively collected at four different 2-years periods over a 30 years time (1980–81 to 2010–11). Information about patient gender, age at injury, injury place and mechanism (s), injured structures, duration of hospital stay, number of out patient visits and rehabilitation visits as well as social status was collected. The injuries were classified with the Modified Hand Injury Severity Score (MHISS).

**Results:**

Most injured patients were men (72%) and the number of patients who reported to be healthy significantly decreased (67% to 18%) during the study period. The number of injuries increased over the study period (*n* = 24 to *n* = 83/2-year period). Outside home was the most common injury place and a saw or a fall was the most frequent injury mechanism. Several fingers were most often injured. The majority of the injuries were classified to be Minor or Moderate (MHISS) and a fracture was the most common injured structure.

**Conclusions:**

We found an increased number of hand injuries over a 30-year period in combination with a decrease in patients reported health treated at a hand surgery ward. Further studies regarding hand trauma in the elderly population will be valuable for future prevention and rehabilitation of this patient group.

## Background

Hand surgery literature lacks specific information regarding hand injuries in the elderly population. The only information is about replantation in the elderly showing no more perioperative complications or mortality in patients over age 65 years compared with those under the age of 65 years [1]. In the age group over 65 years of age, more women suffer from a hand injury, usually a fracture, than men [2].

There has been an increase in mean age in western world, which will lead to a higher proportion of elderly people and an increase in other types of injuries. Thus, one may suspect that also hand injuries have increased [3]. It has been shown that about 25% of all trauma patients, who arrive to an emergency unit, are older than 65 years of age [4]. The mortality rate is higher in older trauma patient, mainly due to high co-morbidity [4]. More than half of the patients suffer from hypertension and about a third suffer from heart disease (Thompson et al.). Falls are the most common injury mechanism and stands for about 75% of all traumas [3]. The fall trauma cause social problems and economical consequences for both the individual and the society [5, 6]. Injuries to the extremities count for about half of the injuries in the elderly patients and arm and hand injuries are a quarter of those injuries [7]. Many factors influence the impact of the trauma in the elderly population; weakness, bad physics, chronic diseases, balance difficulties, decreased reaction and decreased cognitive capacity [3, 8].

In this study, we retrospectively reviewed patients older than 65 years of age over a 30 years period with a hand injury admitted and treated as in-patients at the Department of Hand Surgery in Malmö, Sweden.

## Methods

Medical records were retrospectively reviewed for 216 included patients; 155 men and 61 women, aged older than 65 years of age and admitted with a hand injury as an in-patient in our department. The patients were collected from 2 years periods from the years 1980–81, 1990–91, 2000–01 and 2010–11 with the intention to provide detailed information about the hand injuries in the population as described.

The department has an uptake area of 1.6 to 1.9 million inhabitants over the study period for traumatic hand injuries. All severe and most major and moderate hand trauma cases are treated as in-patients and this has not changed over the study period. All hand injuries except superficial lacerations, minor contusions or sprains from the towns of Malmö and Lund are treated at the department. In the rest of the uptake area all severe and major hand injuries are treated in the department and has not changed during the study period. The clinical practise and the type of injuries treated during the study time have not changed. Distal radius fractures are not included, though they are treated at the orthopaedic departments.

Data about the gender, age at injury, injury place and mechanism, injured structures, duration of hospital stay, number of out patient visits and rehabilitation visits and social status are presented in Table 1 for the total number of patients and for each year group. The patient injuries were classified according to the Modified Hand Injury Severity Score (MHISS) and can be divided in four severity grades depending on the final score (0–20 = Minor, 21–50 = Moderate, 51–100 = Severe and > 100 = Major) [9].

**Table 1:** Characteristics for 216 patients with a hand injury at different time periods

	1980-1981 *n*=24	1990-1991 *n*=42	2000-2001 *n*=67	2010-2011 *n*=83	Total *n*=216	*P*-value
Age at injury	73(66-86)	74(66-86)	76(66-93)	73(66-90)	74(66-93)	0.067
(Years)	SD 6.1	SD 6.1	SD 7.1	SD 6.0	SD 6.5	
Gender F/M (n/%)	9(38) / 15(62)	13(31) / 29(69)	22(33) / 45(67)	17(20) / 66/80	61(28) / 155(72)	0.228
MHISS	66(2-248)	57(4-322)	36(2-300)	46(4-168)	48(2-322)	0.012^a^
	SD 74.1	SD 61.8	SD 52.8	SD 40.6	SD 54.2	
MHISS groups (n/%)						
Minor	6(25)	16(38)	35(53)	28(37)	85(41)	0.015^b^
Moderate	11(46)	10(24)	19(29)	21(27)	61(29)	
Major	5(21)	8(19)	6(9)	8(10)	27(13)	
Severe	2(8)	8(19)	6(9)	20(26)	36(17)	
Missing values	0	0	1	6	7	
Place of injury						
Home	6(25)	16(38)	27(42)	28(34)	77(36)	0.400
Out of home	15(62)	23(55)	34(52)	52(63)	124(58)	
Other	3(13)	3(7)	4(6)	3(3)	13(6)	
Missing values	0	0	2	0	2	
Mechanism of injury						
Fall	5(21)	11(26)	12(18)	10(12)	38(18)	0.069
Saw	5(21)	17(40)	10(15)	25(30)	57(26)	
Knife	3(12)	2(5)	12(18)	6(7)	23(11)	
Machine	2(8)	2(5)	6(9)	10(12)	20(9)	
Other	9(38)	10(24)	26(39)	32(39)	77(36)	
Missing values	0	0	1	0	1	
Type of injury						
Fracture	3(13)	11(26)	16(24)	14(17)	44(20)	0.005 ^c^
Tendon	2(8)	4(10)	11(16)	20(24)	37(17)	
Nerve	0(0)	1(2)	5(8)	11(13)	17(8)	
Combination	7(29)	16(38)	10(15)	10(12)	43(20)	
Other	12(50)	10(24)	25(37)	28(34)	75(35)	
Missing values	0	0	0	0	0	
Anatomical						
Finger	4(17)	10(24)	23(35)	20(25)	57(27)	0.120
Multiple	8(33)	19(45)	12(18)	26(33)	65(31)	
Thumb	5(21)	7(16)	8(12)	17(21)	37(17)	
Vola/Dorsum	4(17)	4(10)	8(12)	8(10)	24(11)	
Other	3(12)	2(5)	15(23)	9(11)	29(14)	
Missing values	0	0	1	3	4	
Comorbidity						
None(Healthy)	16(70)	23(56)	27(42)	15(18)	81(38)	<0.001^d^
Cardiovascular	3(13)	7(17)	15(23)	33(40)	58(28)	
Combination	1(4)	3(7)	17(27)	20(25)	41(20)	
Other	3(13)	8(20)	5(8)	14(17)	30(14)	
Missing values	1	1	3	1	6	
Charlson Comorbidity Index	3.04 SD 0.81	3.29 SD 1.06	3.94 SD 1.22	4.12 SD 1.39	3.78 SD 1.28	<0.001^e^
Ward stay (Days)	4(1-28) 7.0 SD 7.3	2(1-24) 3.4 SD 3.9	2(1-22) 2.8 SD 3.1	2(1-21) 2.8 SD 3.6	2(1-28) 3.4 SD 4.3	0.002^f^
Out patient visits (Days)	3(0-8) 3.3 SD 2.1	4(0-12) 4.0 SD 2.7	5(0-13) 4.6 SD 3.1	4(0-11) 3.5 SD 2.5	3(0-13) 4.0 SD 2.7	0.104
Handrehab visits (Days)	0 0 0	0(0-13) 1.2 SD 2.6	0(0-11) 1.8 SD 2.9	3(0-24) 4.3 SD 5.1	0(0-24) 2.4 SD 4.0	<0.001^g^

Data is presented for the total population and also the data for each of the different time periods. Data are given as number and percentage or as median (min-max) and mean with standard deviation (SD). *P*-values are given based on Kruskal- Wallis

The significant differences, based on Mann Whitney, for “a” is between 1990 and 91 and 2000–01 and between 2000 and 01 and 2010–11, for “b” is between 2000 and 01 and 2010–11, for “c” between 1990 and 91 and 2000–01, for “d” between 1990 and 91 and 2000–01 and between 2000 and 01 and 2010–11, for “e” between 1980 and 81 and 2010–11 and between 1990 and 91 and 2010–11, for “f” between 1980 and 81 and 1990–91 and for “g” between 1980 and 81 and 1990–91 and between 2000 and 01 and 2010–11

No hand rehab visits was registered in 1980–81

### Comorbidity

The comorbidity was grouped into four groups: a) No (Healthy) - none reported disease or medication, b) Cardiovascular, i.e. having hypertension treated, had a myocardial infarction or other cardiovascular disease, c) Combination, e.g. combination of cardiovascular disease and diabetes, cardiovascular and malignant diseases or other combinations, d) Others, e.g. diabetes, depression, malignant diseases or other. We have also classified the comorbidity with the Charlson Comorbidity Scale [10, 11].

### Place of injury and cause of injury

The place of injury was divided into three groups: a) Outside - injuries that took place outside the apartment or house; b) Home - injuries taking place in the apartment or house and c) Others - i.e. injuries taking place in institutions, at work or other places. The cause of injury was grouped into five groups: a) Saw; b) Fall; c) Knife; d) Machine and e) Other (e.g. dog bite, crush, glass and burn).

### Injured part

The injured part is given as a) Multiple fingers, b) Finger, c) Thumb, d) Volar/Dorsum or e) Other (wrist and forearm). No distal radius fractures are included in this study. The type of injury was divided into five groups, a) Fractures, b) Tendon, c) Nerve, d) Combination and e) Others (e.g. lacerations, distal amputation).

### Statistics

All data are presented as median (min-max) if otherwise not stated. The Chi-square method was used to analyse differences between the various years and nominal variables and the Kruskal-Wallis test, with Mann-Whitney test as post hoc tests, to analyse differences between numeric variables and the various years. The Spearman test was used to make correlations with years and e.g. number of ward days. A *p*-value less than 0.05 were considered significant.

## Results

### Incidence, social status, sex and age

Data are presented in Table 1 for the total number of patients and for each time period. An increasing number of patients were seen over the time period. Most of the patients were men, 62% - 80%, depending on which year the injury took place. The median age did not significantly vary. The social status varied over the investigated time period. However, the trend was that more injured patients were married and fewer were living in an institution.

### Comorbidity

Only three patients had diabetes mellitus as a single disease and therefore we have no specific group for these patients. Eleven patients reported to have diabetes in combination with other diseases. The number of patients who reported to be healthy decreased significantly during the study period and were 70% in 1980–81 and 18% in 2010–11. The Charlson Scale showed at the same time that the comorbidity significantly increased over the time period (Table 1). The most common co-morbidity was cardiovascular disease.

### Place of injury

In 214 (99%) patients, we were able to analyse where the injury took place. The most common place to sustain an injury was outside home (*n* = 124, 58%). There was no significant difference in MHISS values or age depending on the injury place. Even if all patients were over the age of 65 years (i.e. age of retirement) some patients were still in the work market and seven (3%) patients of these had an injury at work. They had a median age of 68 years (66–74) and a MHISS score of 24 (6–153).

### Cause of injury

In 215 (99%) patients, we had information about the injury mechanism. The most common injury mechanism was an injury by saw (*n* = 57, 27%) or a fall (*n* = 38, 18%). Twenty-four patients had a knife injury and five of these were due to a suicide attempt.

### Injured part

We had information about which part of the upper extremity that was injured in 212 (98%) patients. Most frequently, multiple fingers were injured (*n* = 31%), followed by a single injured digit (*n* = 57, 27%) and the thumb (*n* = 37, 17%). Forearm injuries were rare (*n* = 8, 4%).

### Type of injury

The injuries were classified into five groups according to the type of injury. There was a significant difference in the type of injury over the time period (*p* < 0.005). Nerve and tendon injuries increased over the study period. Overall the most common injury was a fracture, except the group “Other”, which consisted of a mix of injuries. In the group with tendon injuries, an extensor tendon injury (*n* = 21) was more common than a flexor tendon injury (*n* = 16). In the group with “Other” injuries, there were 38 lacerations (18%) and 25 amputations (12%). The most common amputation was at the level of the DIP joint or distally (n = 16, 8%). Between the middle phalanx and the MCP joint only nine (4%) patients with an amputation were found.

### MHISS

The most common injury severity groups according to MHISS were Minor and Moderate injuries. There was a significant difference in the severity classification between the years (*p* = 0.015). The percentage of injuries classified as Severe increased over the time period from 8% to 24%. Younger retired had higher MHISS than older retired (*p* = 0.001).

Patients who needed to stay more than two weeks in the ward had a more severe injury (median MHISS 126(18–207)). We found a significant correlation between MHISS and the number of ward days (*p* < 0.001). Over 1/3 of the patients (*n* = 82) had a Minor or Moderate injury. However, they still consumed a significant amount of healthcare measured as number of days in ward, number of out patient visits and rehabilitation visits.

### Time of injury

Most injuries took place between Monday and Friday and with the lowest number of injuries on Sunday. The monthly fluctuation showed three peaks, April–May, August and November.

### Frequencies of treatment

All patient were treated as in-patients. The majority of patients (75%) only stayed for three days or less. Only five (2%) patients needed to stay more than two weeks. No correlation was found between age and the number of ward days (*p* = 0.81, corr. Coeff. = − 0.016). A total number of 103 patients (54%) were seen at the outpatient clinic three times or less. However, 12 patients (6%) were never followed up in the outpatient clinic. The younger patients consumed significantly more outpatient visits than the older patients (*p* = 0.029). One hundred (47%) of the patients needed hand therapy, physiotherapy and/or occupational therapy. Of these, 20 (20%) patients had one session and only 13 (10%) patients needed more than nine sessions. The younger patients consumed significantly more rehabilitation visits than the older patients (*p* = 0.018). The patients consumed more hand rehabilitation during the later part of the studied time period.

The patients with Minor and Moderate injuries were 144 and 77% stayed up to three days in the ward and 60% had between one and three outpatient visits. Eighty-two (57%) of these patients did not need any rehabilitation contact.

## Discussion

We found an increasing number of patients with hand injuries needing admission to the ward over the time period. From 1980 to 81 to 2010–11 the number of patients with injuries increased four times, and the increasing number of older people in the uptake area may to some extent explain this increase. However, during the same time period the number of people over 65 years of age only increased with 2% in the uptake area (www.scb.se). The approximated incidence for the injuries in the group of inhabitants over the age of 65 years in the uptake are increased from 9.6 injuries/100000 inhabitants and year to 25.2 injuries/100000 inhabitants and year. The number of patients, and also the incidence, only reflects those patients who were admitted to the ward for surgery. We selected an interrupted time series with the purpose to evaluate details about the hand injuries, such as e.g. HISS indicating the severity of the injury, in the population and not available from other sources than the present patient folders. Those patients who only needed treatment as day surgery patients or only received a plaster or a wound dressing are not included in this study, which may be a limitation of the study. Further studies, including all patients treated for a hand injury, need to be investigated to get a precise incidence of all types of hand injuries in the elderly population, but such statistics was not available at our hospital for the time periods 1980–81 and 1990–91. There could be confounding factors that influence the observed increase. However, no major change has been done during the study period regarding admission criteria to our department. These elderly patients, with a significant hand injury and also sometimes complicating comorbidity, is principally not treated as day-surgery patients. The difference between the increase in population and the increase in hand injuries in this population is huge (2% compared to 160%) and even if there are confounding factors that we do not know, the probability for an increase in important hand injuries requiring a stay at the ward must be suspected.

In the society, the elderly people represent an increasing part of the population, as the population live longer and the mortality even among the elderly decreases [12, 13]. A United Nation report shows that over the next decades the highest population increase will be in the age group over 60 years of age and the prognosis is for all parts of the world independent of level of development (United Nations 2015. World Population Prospects. The 2015 revision)

Studies have found a change in habit with a more active life also after retirement and may explain an increasing number of injuries [14].

We did not find any increase in age at injury over the study period. The younger elderly consumed more outpatient visits and rehabilitation visits, which can be explained by a higher MHISS scores in these patients (*p* = 0.001). The finding that more men were injured than women during the study period is in conflict with earlier reports [2]. However, we have only included patients treated after being admitted to the ward, which might explain the gender difference.

The comorbidity, measured with the Charlson Scale, significantly increased over the years, which might be due to an increase in prescription of medications as seen in other countries [15]. Those who reported to be healthy also decreased over the study period. The number of elderly subjects with a disability has decreased from the 1980:is and forward, which is shown in several studies [16–19]. The information regarding comorbidity in our study was received from the patient notes. One cannot exclude the possibility that there could be missing information regarding the comorbidity and thus an underestimation of these figures, which is a limitation of the study. In spite of needing treatment for different diseases, the subjects managed daily living without support. However, a minor hand injury may cause a higher degree of disability in an older person who then may need support from the society to be able to manage their activities of daily living [20].

A fall that results in a distal radius fracture or a hip fracture is common in the older population [21]. Elderly patients have a higher risk for complications following surgery for distal radius fractures compared to non-surgical treatment without any major difference in outcome [22]. The overall age specific number of fractures have been found to increase with age in women, but has been shown to be stable in men [23]. Also, in our study, fractures from falls were a common injury mechanism and represented around 20% of the injuries. However, a recent study has shown a decrease in distal radius fractures in women during 2001 [24]. The number of fall accidents decreased during the study period, but we found no decrease in the number of fractures in the hand over the time period. However, only those fractures that needed surgery, as an in-patient, is included. Further investigations, including patients with closed fractures treated with a plaster as outpatients, need to be performed.

A minority of the subjects were still working after retirement and had an injury at work. According to official Swedish statistics (www.scb.se), about 7% of the population between 66 and 74 years are working, with more men (10%) than women (4%) being active at the labour market. However, that cannot explain the fact that most patients were injured during Monday to Friday. Interestingly, there were peaks in April–May, August and November, which may indicate injuries related to outdoor activities (see e.g. Table 1); information needed for preventive work. Thus, the data can be used in prevention work also in this older population; work that has been efficient in other age groups still active in the labor market.

The study only includes patients needing surgery as an in-patient and the number of patients treated as outpatients are not known, which is a limitation in the study. Patients, who were treated as outpatients from the year periods 1980–81 and 1990–91, could not be identified in the hospital register. However, all patients needing a ward stay, which include all more severe injuries, were evaluated. The indication for admitting a patient to the ward has not changed during the study period at our department.

Studies have shown that preventive programs, all including some sort of physical training, have been found to decrease the number of falls in orthopaedic patients [25]. Few studies have reported the effect of prevention on hand injuries. Those studies found in the literature, only give information on industrial hand-injury prevention [26], give advice on how to prevent hand injuries from specific machines or tools [27, 28] or trying to make a more general country wide campaign [29]. There is no specific hand injury preventive study for the elderly population. This study is a retrospective epidemiologic study aiming at collecting as much information about the hand injuries in this population group and building a base for further studies. With this study, we do not have enough information to intervene with specific hand injury prevention guidelines. From an earlier study on hand injuries from wood-cutters, we found that most of the injuries occurred because the equipment was not used in the correct way [30]. In this study, many injuries were saw injuries and some of them might have been do to incorrect use. The recommendation is to read the instruction carefully and use the equipment accordingly.

Further studies, also including non-admitted patients, regarding hand trauma in the elderly will be valuable since the number of elderly will increase and they may need other types of decision-making regarding treatment and rehabilitation.

## Conclusions

We found an increased numbers of hand injuries over a 30-year period in combination with an increasing comorbidity treated at a hand surgery ward. Further studies regarding hand trauma in the elderly population will be valuable for future prevention programs and the rehabilitation of this patient group.

## Abbreviations

MHISS: Modified Hand Injury Severity Score

## References

[1] Barzin A, Hernandez-Boussard T, Lee GK, Curtin C (2011). Adverse events following digital replantation in the elderly. J Hand Surg Am.

[2] Rosberg HE, Dahlin LB (1997). Epidemiology of hand injuries in a middle-sized city in southern Sweden: a retrospective comparison of 1989 and. Scandinavian journal of plastic and reconstructive surgery and hand surgery / Nordisk plastikkirurgisk forening [and] Nordisk klubb for handkirurgi 2004.

[3] Bonne S, Schuerer DJ (2013). Trauma in the older adult: epidemiology and evolving geriatric trauma principles. Clin Geriatr Med.

[4] Keller JM, Sciadini MF, Sinclair E, O'Toole RV (2012). Geriatric trauma: demographics, injuries, and mortality. J Orthop Trauma.

[5] Hartholt KA, Polinder S, Van der Cammen TJ, Panneman MJ, Van der Velde N, Van Lieshout EM, Patka P, Van Beeck EF (2012). Costs of falls in an ageing population: a nationwide study from the Netherlands (2007-2009). Injury.

[6] Roudsari BS, Ebel BE, Corso PS, Molinari NA, Koepsell TD (2005). The acute medical care costs of fall-related injuries among the U.S. older adults. Injury.

[7] Kara H, Bayir A, Ak A, Akinci M, Tufekci N, Degirmenci S, Azap M (2014). Trauma in elderly patients evaluated in a hospital emergency department in Konya, Turkey: a retrospective study. Clin Interv Aging.

[8] Kannus P, Sievanen H, Palvanen M, Jarvinen T, Parkkari J (2005). Prevention of falls and consequent injuries in elderly people. Lancet.

[9] Urso-Baiarda F, Lyons RA, Laing JH, Brophy S, Wareham K, Camp D (2008). A prospective evaluation of the modified hand injury severity score in predicting return to work. Int J Surg.

[10] Wang HY, Chew G, Kung CT, Chung KJ, Lee WH (2007). The use of Charlson comorbidity index for patients revisiting the emergency department within 72 hours. Chang Gung Med J.

[11] Charlson ME, Pompei P, Ales KL, MacKenzie CR (1987). A new method of classifying prognostic comorbidity in longitudinal studies: development and validation. J Chronic Dis.

[12] Ahlbom A, Drefahl S, Lundstrom H (2010). The aging population. Continuing increase of average longevity is a controversial and exciting question. Lakartidningen.

[13] Vaupel JW (2010). Biodemography of human ageing. Nature.

[14] Marmot M, Banks J, Blundell R, Lessof C, Nazroo J (2003). Health, wealth and lifestyles of the older population in England: THE 2002 ENGLISH LONGITUDINAL STUDY OF AGEING.

[15] Charlesworth CJ, Smit E, Lee DS, Alramadhan F, Odden MC (2015). Polypharmacy among adults aged 65 years and older in the United States: 1988-2010. J Gerontol A Biol Sci Med Sci.

[16] Crimmins EM (2004). Trends in the health of the elderly. Annu Rev Public Health.

[17] Manton KG, Gu X, Lamb VL (2006). Change in chronic disability from 1982 to 2004/2005 as measured by long-term changes in function and health in the U.S. elderly population. Proceedings of the National Academy of Sciences of the United States of America.

[18] Moe JO, Hagen TP (2011). Trends and variation in mild disability and functional limitations among older adults in Norway, 1986-2008. European journal of ageing.

[19] Aijanseppa S, Notkola IL, Tijhuis M, van Staveren W, Kromhout D, Nissinen A (2005). Physical functioning in elderly Europeans: 10 year changes in the north and south: the HALE project. J Epidemiol Community Health.

[20] Shapiro MJ, Partridge RA, Jenouri I, Micalone M, Gifford D (2001). Functional decline in independent elders after minor traumatic injury. Academic emergency medicine: official journal of the Society for Academic Emergency Medicine.

[21] Tsuda T (2017). Epidemiology of fragility fractures and fall prevention in the elderly: a systematic review of the literature. Curr Orthop Pract.

[22] Lutz K, Yeoh KM, MacDermid JC, Symonette C, Grewal R (2014). Complications associated with operative versus nonsurgical treatment of distal radius fractures in patients aged 65 years and older. The Journal of hand surgery.

[23] Rosengren BE, Karlsson M, Petersson I, Englund M (2015). The 21st-century landscape of adult fractures: cohort study of a complete adult regional population. Journal of bone and mineral research : the official journal of the American Society for Bone and Mineral Research.

[24] Brogren E, Petranek M, Atroshi I (2007). Incidence and characteristics of distal radius fractures in a southern Swedish region. BMC Musculoskelet Disord.

[25] Karlsson MK, Vonschewelov T, Karlsson C, Coster M, Rosengen BE (2013). Prevention of falls in the elderly: a review. Scandinavian journal of public health.

[26] Smith BL (1987). An inside look: hand injury-prevention program. The Journal of hand surgery.

[27] Master D, Piorkowski J, Zani S, Babigian A (2008). Snowblower injuries to the hand: epidemiology, patterns of injury, and strategies for prevention. Ann Plast Surg.

[28] Eriksson M, Karlsson J, Carlsson KS, Dahlin LB, Rosberg HE (2011). Economic consequences of accidents to hands and forearms by log splitters and circular saws: cost of illness study. Journal of plastic surgery and hand surgery.

[29] Bellemere P, Guimberteau JC (2013). Experience of a national campaign for hand trauma prevention in France. Handchirurgie, Mikrochirurgie, plastische Chirurgie : Organ der Deutschsprachigen Arbeitsgemeinschaft fur Handchirurgie: Organ der Deutschsprachigen Arbeitsgemeinschaft fur Mikrochirurgie der Peripheren Nerven und Gefasse.

[30] Eriksson M, Karlsson J, Steen Carlsson K, Dahlin LB, Rosberg HE (2011). Economic consequences of accidents to the hands and forearms by log splitters and circular saws: cost of illness study. J Plast Surg Hand Surg.

